# Sexual Assault Nurse Examiners Lead to Improved Uptake of Services: A Cross-Sectional Study

**DOI:** 10.5811/westjem.59514

**Published:** 2023-08-11

**Authors:** Meredith Hollender, Ellen Almirol, Makenna Meyer, Heather Bearden, Kimberly A. Stanford

**Affiliations:** * University of Chicago, Pritzker School of Medicine, Chicago, Illinois; † Chicago Center for HIV Elimination, Chicago, Illinois; ‡ University of Chicago, Section of Emergency Medicine, Chicago, Illinois

## Abstract

**Introduction:**

Sexual Assault Nurse Examiners (SANE), who are trained to provide comprehensive and compassionate specialty care to sexual assault survivors, are increasingly used in the emergency department (ED), but there is little published literature to support their benefit. In this study we aimed to compare services offered and received by sexual assault survivors in the ED when care was provided by a SANE vs those with traditional care teams, hypothesizing that SANE utilization will be associated with improved uptake of recommended services.

**Methods:**

This was a retrospective review examining all patient encounters in which a sexual assault was disclosed in a large, urban, adult ED between June 1, 2019–June 30, 2022. We extracted timeline information from the ED encounter, demographic information, resources offered to and accepted by the patient, clinical care data, and continuity of care data from the medical record. We used unadjusted and adjusted analyses to compare patient demographics and services offered and accepted between SANE and non-SANE encounters.

**Results:**

We included a total of 182 encounters in the analysis, of which 130 (71.4%) involved SANEs. Demographics were similar between groups, except there was a larger proportion of cisgender men in the non-SANE group (14.0% vs 5.5%), and the timing of visits differed, with non-SANE visits more common during the overnight shift. All recommended testing, prophylaxis, and resources were offered more frequently during SANE visits, and all but one were more frequently accepted by patients during SANE visits, although not all comparisons reached statistical significance.

**Conclusion:**

Patients who received care from a SANE were more often offered recommended services and resources and more frequently accepted them. Making SANE care available at all times to these vulnerable patients would both improve patient outcomes and allow hospitals to meet required quality metrics. States should consider expanding legislation to encourage and fund SANE coverage for all hospitals to support access to vital resources in the ED for survivors of sexual assault.

Population Health Research Capsule What do we already know about this issue?

*Few studies have examined the impact of Sexual Assault Nurse Examiners (SANE) on the patient experience, or evaluated their effects on delivery of recommended services.*

What was the research question?

*Were sexual assault survivors more likely to receive recommended healthcare services in the ED if they were cared for by a SANE?*

What was the major finding of the study?

*Patients cared for by a SANE were more likely to be offered advocate services (P < 0.05), medical forensic exam kits (P < 0.05), and resource packets (P < 0.05).*

How does this improve population health?

*Sexual assault survivors cared for by a SANE are more likely to receive recommended treatment in the ED, which may have major impacts on long-term outcomes for them.*

## INTRODUCTION

Sexual assault (SA) is a major public health issue that affects people of all socioeconomic and cultural backgrounds. Each year in the United States, more than 100,000 survivors of SA seek care in the emergency department (ED).^1^ Understanding that survivors of SA are a vulnerable group with unique acute and chronic care needs, there have been recent moves toward implementing legislation that will help support comprehensive and compassionate hospital care for survivors, including the federal Survivors’ Bill of Rights Act of 2016, the Illinois Sexual Assault Survivors Emergency Treatment Act (SASETA) amended in 2019, and the No Surprises for Survivors Act, introduced in 2022.^2^^–^^4^ These laws aim to protect SA survivors from financial ruin after their hospital visit, ensure medical forensic kits are processed in an efficient and timely manner, establish care guidelines and reporting systems for hospitals that treat SA survivors, and promote the provision of comprehensive, trauma-informed care.

The goal of these initiatives is to address inequities in access to care faced by survivors of SA, among whom the most vulnerable members of society—particularly young, socioeconomically disadvantaged women— are disproportionately represented.^5^^–^^7^ Survivors face many barriers to receiving optimal care, with fewer than one in five US hospitals providing all 10 metrics of what is considered “comprehensive medical care management” for SA survivors in the ED.^6^ One proposed mechanism to address this gap is to use specially trained Sexual Assault Nurse Examiners (SANE).

Sexual Assault Nurse Examiner programs were developed in 1976 to augment training, address concerns of physicians about caring for survivors of sexual assault, decrease long ED wait times, and support sensitive and socially competent care.^8^ Many emergency physicians and nurses feel unprepared and uncomfortable providing some or all necessary patient care in cases of SA.^9^ They may also face significant challenges due to the unique ED environment, including crowding, caring for multiple patients simultaneously, or needing to care for patients who require immediate attention. Earlier studies have shown that when SANEs provide care, they do so in a compassionate, respectful, and safe manner that is associated with feelings of confidence and relief in survivors.^10^^,^^11^ The SANEs are able to build a unique, trusting relationship with their patients that is focused on providing SA survivors with control and choices surrounding their care decisions.^12^

Sexual Assault Nurse Examiner programs are increasingly common, and there is rising awareness of the importance of specialized SANE training; however, there is still a nationwide shortage of SANEs.^13^ Rural areas have few SANEs and are less likely than urban areas to have 24-hour continuous SANE coverage.^14^ Even in urban areas, 85.5% of nurses indicate that they are not SANE-trained, yet they have cared for SA survivors in their healthcare institution.^13^ While some studies have examined the impact of SANEs on the patient experience, few have evaluated the effects of SANE care on quality metrics in terms of the actual delivery of recommended services and resources.

We conducted this study at a large, urban, tertiary care hospital with a Level I trauma center. Approximately 60–80 adult survivors of SA visit our ED annually. In this ED, a SANE is unavailable in the adult ED approximately 20-30% of the time, mostly overnight. Care for a SA survivor is always provided by a SANE, if available; if not, then care is provided by a physician or non-physician clinician and a registered nurse, which is the standard of care. During a visit for SA, patients are offered a medical forensic examination kit, if appropriate, which can be used as evidence should a case go to trial, testing and prophylaxis for HIV and other sexually transmitted infections (STI), a pregnancy test, and emergency contraception. They should also have the opportunity to speak with police, a SA advocate, and social services, and access other hospital resources as needed, such as behavioral health support or safe housing options, and they are given a packet of post-visit resources before discharge. Both SANEs and registered nurses have access to a checklist of these items that should be completed during the ED visit. This study compared the rate at which these services were offered or accepted between encounters in which care was provided by a SANE vs those with traditional care teams, hypothesizing that SANEs would be more likely to offer services, and that their patients would be more likely to accept them. Increased uptake of recommended services with SANE care would support the adoption and expansion of SANE programs, which would ultimately address disparities in care faced by SA survivors.

## METHODS

### Study Design

This was a retrospective review of all adult patient encounters in which a SA was disclosed (defined as a triage chief complaint or ED diagnosis code for SA) in the ED between June 1, 2019–June 30, 2022. Encounters were included even if the patient left before the visit was considered complete, provided any services had been offered. For patients who were included more than once (i.e., had two instances of SA during the study period), each encounter was considered an independent event, as the details of the assault and the ED encounter were unique. This study was approved by our institutional review board.

### Measures

Data extracted from the electronic health record (EHR) included the following: demographics (e.g., age, gender, race/ethnicity); the time the patient was admitted to the ED; days since incident reported; clinical care data (e.g., STI testing and prophylaxis, forensic kit collection, pregnancy testing and provision of emergency contraceptives); and continuity of care data (e.g., linkage to primary care or mental health resources). Encounter time was divided according to shift times in the ED, with 6:30 am–2:30 pm considered the morning, 2:30 pm–10:30 pm considered the afternoon, and 10:30 pm–6:30 am considered overnight.

The primary outcomes of this study were the proportions of patients offered and accepting services when cared for by a SANE or non-SANE team. In certain cases, for which a service was not applicable during the ED visit (e.g., already completed elsewhere, too much time passed since the incident, or pregnancy testing for individuals without a uterus), these were removed from the denominator of services offered. If an applicable service was not explicitly documented to have been offered or accepted in the EHR, it was considered not offered or not accepted for the purposes of the analysis. If a service was partially accepted (e.g., prophylaxis for gonorrhea and chlamydia but not hepatitis), the outcome (e.g., STI prophylaxis) was considered accepted in the analysis.

### Statistical Analyses

We compared patient demographics and the proportion of patients offered and accepting services to identify differences between SANE and non-SANE evaluated patients, using a *t*-test for continuous variables and chi-square (χ2) test or the Fisher exact test for categorical variables. We used logistic regression to calculate odds ratios between groups, adjusting for patient arrival time as a potential confounder in the model. Offering the medical forensic kit, accepting the discharge resource packet, and accepting the social work consult were excluded from the logistic regression analysis due to the presence of zero responses. Differences were considered significant at *P* ≤ 0.05. We performed all statistical analyses using R version 4.2.1. (R Core Team, R Foundation for Statistical Computing, Vienna, Austria).

## RESULTS

### Participant Characteristics

Over the three-year study period, we identified 182 adult ED encounters for SA, including 177 unique individuals, five of whom presented on two separate occasions for SA. Of all encounters, 130 (71.4%) received care from a SANE, while 52 (28.6%) received the standard of care with a physician/nurse team (Table 1). Cisgender women (90.4%) and non-Hispanic Black individuals (82.7%) represented the majority of encounters, with a mean age of 30 years (range 18–79). Demographics were similar between the two groups; however, the non-SANE group had more cisgender men than the SANE group (14.0% vs 5.5%) and no transgender individuals. The groups differed by time of patient arrival, with a larger proportion of SANE encounters (48.5%) in the afternoon, and the largest proportion of non-SANE encounters (50.0%) during the overnight shift (*P* < 0.01). Both SANE and non-SANE groups presented to the ED within similar time frames after the assault (mean 1.16 days, SD 1.44, non-SANE vs mean 1.28 days, SD 1.72, SANE; *P* = 0.65). Additionally, there was a significant difference in the number of patients who left before treatment was complete (15.4% non-SANE vs 2.3% SANE, *P* < 0.01).

**Table 1.** Demographics of emergency department patient encounters for sexual assault from June 1, 2019–June 30, 2022, by type of care team.

	All encounters (n = 182)		SANE (n = 130)		Non-SANE (n = 52)		*P*-value
	n	(%)	n	(%)	n	(%)	
Age (mean, SD)	30.2	(13.1)	30.1	(13.5)	30.6	(12.3)	0.81
Gender^+^							0.11
Female	160	(90.4%)	117	(92.1%)	43	(86.0%)	
Male	14	(7.9%)	7	(5.5%)	7	(14.0%)	
Transgender/Non-binary	3	(1.7%)	3	(2.4%)	0	(0.0%)	
Race/Ethnicity^+^							0.83
Non-Hispanic White	14	(8.4%)	10	(8.1%)	4	(9.3%)	
Non-Hispanic Black	138	(82.7%)	101	(81.5%)	37	(86.0%)	
Hispanic	9	(5.4%)	8	(6.5%)	1	(2.3%)	
Other	6	(3.6%)	5	(4.0%)	1	(2.3%)	
Patient arrival time^^^							<0.01^*^
Morning	48	(26.4%)	32	(24.6%)	16	(32.0%)	
Afternoon	73	(40.1%)	63	(48.5%)	10	(18.0%)	
Overnight	61	(33.5%)	35	(26.9%)	26	(50.0%)	
Days since incident (mean, SD)	1.19	1.51	1.16	1.44	1.28	1.72	0.65

*SANE*, Sexual Assault Nurse Examiner.

* Indicates a *P*-value ≤ 0.05; Fisher tests were conducted due to the small cell counts.

+ Gender (n = 177) and race/ethnicity (n = 177), given that five individuals had two encounters.

^ Patient arrival times were categorized into the following: morning 6:30 am–2:30 pm; afternoon 2:30 pm–10:30 pm; and overnight 10:30 pm–6:30 am.

Missing values for race/ethnicity (n = 15, 8%) and days since incident (n = 7, 4%).

### Resources, Medical Care, and Services Offered and Accepted

While not all differences were statistically significant, every type of recommended resource or care studied was offered in a higher proportion of SANE encounters than non-SANE encounters (Table 2). Significant differences observed in services offered between SANE and non-SANE groups included SA advocate (97.7% SANE vs 89.4% non-SANE; odds ratio [OR] 5.04, 95% confidence interval [CI] 1.16-21.99, *P* = 0.03), medical forensic kit (100% vs 93.6%, *P* = 0.02); pregnancy testing (96.2% vs 86.1%; OR 4.11, 95% CI 1.09-15.54, *P* = 0.05); and discharge resource packet (69.0% vs 48.9%; OR 2.33, 95% CI 1.16-4.65, *P* = 0.03). A higher percentage of the SANE group was offered emergency contraception (94.3% vs 82.4%, *P* = 0.07), although not significant. The proportion of encounters offered safe disposition planning (28.7% vs 21.7%) or a social work consult (33.3% vs 27.7%) was markedly low in both groups, although it is unknown whether this was related simply to lack of documentation around these services.

**Table 2.** Bivariate analysis of services offered and accepted by emergency department patients evaluated after sexual assault from June 1, 2019–June 30, 2022, by type of care team.

	SANE (n = 130)		Non-SANE (n = 52)		*P*-value
	n/N	(%)	n/N	(%)	
Testing and prophylaxis					
HIV testing					
Offered^+^	122/128	(95.3%)	42/46	(91.3%)	0.46
Accepted^+^	113/121	(93.4%)	36/42	(85.7%)	0.20
STI testing					
Offered^+^	124/129	(96.1%)	43/47	(91.5%)	0.25
Accepted^+^	115/123	(93.5%)	37/43	(86.0%)	0.20
Pregnancy testing					
Offered^+^	102/106	(96.2%)	31/36	(86.1%)	0.05^*^
Accepted^+^	94/101	(93.1%)	28/31	(90.3%)	0.70
HIV prophylaxis					
Offered^+^	115/126	(91.3%)	39/44	(88.6%)	0.56
Accepted	87/114	(76.3%)	25/39	(64.1%)	0.20
STI prophylaxis					
Offered^+^	122/129	(94.6%)	41/46	(89.1%)	0.31
Accepted	103/121	(85.1%)	33/41	(80.5%)	0.65
Emergency contraception					
Offered^+^	99/105	(94.3%)	28/34	(82.4%)	0.07
Accepted	65/98	(66.3%)	16/28	(57.1%)	0.50
Services and resources					
Medical forensic kit					
Offered^+^	129/129	(100.0%)	44/47	(93.6%)	0.02^*^
Accepted	114/129	(88.4%)	30/44	(68.2%)	<0.01^*^
Sexual assault advocate					
Offered^+^	127/130	(97.7%)	42/47	(89.4%)	0.03^*^
Accepted	100/127	(78.7%)	26/42	(61.9%)	<0.05^*^
Police report					
Offered to call^+^	119/125	(95.2%)	40/44	(90.9%)	0.29
Report complete	98/119	(82.4%)	27/40	(67.5%)	0.08
Resource packet					
Offered	89/129	(69.0%)	22/45	(48.9%)	0.03^*^
Accepted^+^	88/89	(98.9%)	22/22	(100.0%)	1.00
Social worker consult					
Offered	43/129	(33.3%)	13/47	(27.7%)	0.60
Accepted^+^	43/43	(100.0%)	11/13	(84.6%)	0.05
Safe discharge planning					
Offered	37/129	(28.7%)	10/46	(21.7%)	0.47
Accepted^+^	36/37	(97.3%)	9/10	(90.0%)	0.38
Left before treatment complete	3/130	(2.3%)	8/52	(15.4%)	<0.01

*SANE*, Sexual Assault Nurse Examiner; *STI*, sexually transmitted infections.

* Indicates a *P*-value ≤ 0.05; ^+^Fisher tests were performed due to small cell counts.

For those services documented to have been applicable and offered, the proportion of recommended resources and care accepted in SANE encounters was also higher for every service category except the discharge resource packet, which was comparable between groups (98.9% SANE vs 100.0% non-SANE, *P* = 1.00). A much larger proportion of encounters in the SANE group accepted SA advocate services (78.7% vs 61.9%; OR 2.28, 95% CI 1.07-4.84, *P* = 0.05) and a medical forensic kit (88.4% vs 68.2%; OR 3.55, 95% CI 1.54-8.15, *P* < 0.01). Large differences that did not reach statistical significance were found for several service types, including making a police report (82.4% vs 67.5%), HIV prophylaxis (76.3% vs 64.1%), HIV testing (93.4% vs 85.7%) and STI testing (93.5% vs 86.0%), emergency contraception (66.3% vs 57.1%), and social worker consultation (100% vs 84.6%).

Because SANE encounters occurred more often during the afternoon and non-SANE encounters more often overnight, additional models were created to adjust for the effects of patient arrival time on services offered and accepted (Tables 3 and 4). In the adjusted analysis, SANE encounters were still more likely to offer recommended services such as SA advocates (adjusted [aOR] 5.51, 95% CI 1.26-24.05, *P* = 0.03) and to accept both the advocate services (aOR 2.60, 95% CI 1.22-5.52, *P* = 0.02) and the medical forensic kit (aOR 2.90, 95% CI 1.26-6.66, *P* = 0.02). Of note, after adjusting for arrival time, the higher proportion of SANE encounters completing a police report was significant (aOR 2.63, 95% CI 1.17-5.93, *P* = 0.03). While the non-SANE group had a higher proportion of cisgender men than the SANE group (Table 1), the model was not adjusted for patient gender, as this observed difference did not reach statistical significance.

**Table 3.** Odds ratios of services offered to emergency department patients evaluated after sexual assault from June 1, 2019–June 30, 2022, by type of care team.

	Unadjusted odds ratio	95% Confidence interval	*P*-value	Adjusted odds ratio	95% Confidence interval	*P*-value
Testing and prophylaxis						
HIV testing	2.35	(0.77, 7.24)	0.13	1.93	(0.63, 5.95)	0.27
STI testing	2.33	(0.76, 7.16)	0.14	2.40	(0.78, 7.36)	0.15
Pregnancy testing	1.44	(1.00, 16.96)	0.62	1.41	(0.87, 14.79)	0.64
HIV prophylaxis	1.80	(0.82, 3.95)	0.14	1.87	(0.86, 4.10)	0.14
STI prophylaxis	1.39	(0.55, 3.48)	0.49	2.11	(0.48, 3.04)	0.70
Emergency contraception	1.43	(0.61, 3.38)	0.41	1.61	(0.68, 3.80)	0.29
Services and resources						
Medical forensic kit	3.55	(1.54, 8.15)	<0.01^*^	2.90	(1.26, 6.66)	0.02^*^
Sexual assault advocate	2.28	(1.07, 4.84)	0.03^*^	2.60	(1.22, 5.52)	0.02^*^
Police report	2.25	(1.00, 5.06)	0.05	2.63	(1.17, 5.93)	0.03^*^
Resource packet	N/A			N/A		
Social worker consult	N/A			N/A		
Safe discharge planning	4.00	(0.23, 70.30)	0.34	3.64	(0.21, 63.91)	0.40

*STI*, sexually transmitted infections.

* Indicates a *P*-value ≤ 0.05; reference group = non-Sexual Assault Nurse Examiners. Analyses are adjusted for patient arrival time. Odds ratio analysis could not be calculated for N/A entries due to 0 responses in a single group.

**Table 4.** Odds ratios of services accepted by emergency department patients evaluated after sexual assault from June 1, 2019, through June 30, 2022, by type of care team.

	Unadjusted odds ratio	95% Confidence interval	*P*-value	Adjusted odds ratio	95% Confidence interval	*P*-value
Testing and prophylaxis						
HIV testing	1.94	(0.52, 720)	0.32	2.04	(0.55, 7.58)	0.32
STI testing	2.31	(0.59, 8.99)	0.23	2.04	(0.52, 7.94)	0.33
Pregnancy testing	4.11	(1.09, 15.54)	0.04^*^	3.59	(0.95, 13.55)	0.08
HIV prophylaxis	1.34	(0.44, 4.10)	0.61	1.07	(0.35, 3.26)	0.92
STI prophylaxis	2.13	(0.64, 7.06)	0.22	1.59	(0.48, 5.29)	0.47
Emergency contraception	3.54	(1.05, 11.78)	0.04^*^	3.00	(0.90, 9.97)	0.08
Services and resources						
Medical forensic kit	N/A			N/A		
Sexual assault advocate	5.04	(1.16, 21.99)	0.03^*^	5.51	(1.26, 24.05)	0.03^*^
Police report	1.98	(0.53, 7.39)	0.31	1.87	(0.50, 6.96)	0.37
Resource packet	2.33	(1.16, 4.65)	0.02^*^	1.96	(0.98, 3.93)	0.07
Social worker consult	1.21	(0.58, 2.54)	0.59	1.05	(0.50, 2.19)	0.91
Safe discharge planning	1.45	(0.65, 3.23)	0.36	1.34	(0.60, 2.98)	0.49

*STI*, sexually transmitted infections.

* Indicates a *P*-value ≤ 0.05; Reference group = non-Sexual Assault Nurse Examiners. Analyses are adjusted for patient arrival time. Odds ratio analysis could not be calculated for N/A entries due to 0 responses in a single group.

## DISCUSSION

We found that survivors cared for by a SANE were more often offered the recommended care and resources in every category examined, and they accepted this offer more often for all but one category. The SANEs were significantly more likely to offer a pregnancy test and emergency contraception, and survivors cared for by a SANE were significantly more likely both to be offered and to accept SA advocate services and a medical forensic examination kit. While only a few categories reached statistical significance, this is likely due to the small sample size inherent in studying a relatively uncommon event, and the results of this study suggest major potential benefits from SANE care.

Recent data shows a concerning trend in US ED visits for SA, which have increased more than 1,533.0% from 2006 to 2019.^5^ Young, low-income women are disproportionately represented among survivors of SA.^5^ Survivors may have increased risk for a variety of mental health complications, substance use, and chronic health conditions.^15^^–^^17^ Providing comprehensive and trauma-informed care to survivors of SA in the ED is vital to the long-term outcomes for these vulnerable patients. However, time constraints, crowding, lack of awareness or training in trauma-informed care, and many other challenges of the ED environment can present major obstacles.

Recent federal and state laws have been passed or proposed to try to address this problem, requiring certain standards for all visits for SA. For example, the Illinois SASETA act created universal care and reporting guidelines and requires EDs to have continuous coverage by a SANE or a clinician with equivalent training.^4^ Guidelines from the American College of Emergency Physicians on management of patients presenting after SA emphasize the importance of “specially trained, non-physician medical personnel,” which may include SANEs, and “access to appropriate medical, technical, and psychological support” for patients.^18^ In addition, with the recent increasing popularity of the value-based reimbursement model, there will be financial incentives for hospitals to provide services to SA survivors beyond basic medical care.^19^ Similarly, there may be financial penalties or legal ramifications for hospitals that do not meet these quality metrics in states that have implemented laws like SASETA.

The SANE programs have been proposed to fill these gaps, using dedicated care personnel with specialized training in caring for survivors of SA. These programs have been shown to reduce patient wait times, increase quality of examination and evidence collection, and provide overall comprehensive and compassionate care in a timely manner.^20^ Despite growing evidence and guidelines supporting SANE services, SANE utilization and availability are highly variable. One study found that 35.5% of hospitals had no access to SANE services at all.^13^ Hiring and retaining a specially trained group of nurses to be available at all times, if needed, is expensive and challenging. For hospitals without the means to expand SANE coverage, community efforts have laid the groundwork for telehealth SANE coverage in rural areas.^21^

While there is a growing body of evidence demonstrating the benefits of SANE care, thus far little has been published on the effect of SANE care on quality metrics, which are important both for individual patient outcomes and regulatory and financial reasons. Evidence of an association between SANE care and improved service delivery could encourage expanded support for the development and adoption of SANE programs. Prior studies suggest that specialized training may help nurses approach patients about receiving medical services for a SA in a manner that encourages engagement in care.^11^^,^^22^ When care is provided by a SANE, patients report positive psychological outcomes, such as feelings of empowerment and compassion.^11^^,^^22^

One study found that SANEs go beyond “collecting evidence,” and that “the manner in which it was being done” made a positive impact on patients.^10^ Patients interviewed in that study found that SANEs provided a “clear and thorough explanation of the exam process and findings.”^10^ In the present study, more survivors completed treatment in the ED when care was provided by a SANE. This may reflect the additional training in trauma-informed care or the lack of concurrent clinical duties during SANE care, both of which may lead patients to engage more in their care. Additionally, when services were offered to survivors, they were accepted at much higher rates when offered by a SANE. This likely reflects the way in which the resource was presented or described to the survivor, which certainly could be affected by training and awareness.

Involvement of SANES in care is associated with more medical services provided, more forensic kits collected, and more police reports filed.^23^ The same trend was identified for SANE care in cases of pediatric SA.^24^ In the current study, after adjusting for patient arrival time, police reports were completed during SANE encounters at a significantly higher rate. The police reporting options for patients are complex, there can be significant delays waiting for police to arrive to file a report, and non-SANE nurses may not be familiar with all the options, which may lead to missed opportunities to file a police report.

Non-SANE nurses caring for SA survivors have a checklist of services to offer and, therefore, theoretically should offer these services at the same rate. However, SANEs receive substantial additional training that may afford them a better understanding of the importance of these resources and the skills to discuss them sensitively with a traumatized patient. A SANE-trained nurse may have a more positive attitude toward SA survivors in general.^13^ They may also have more time to talk with the patients, as they are not responsible for any other patient care duties at the same time; or the higher resource acceptance rates among SANE patients may simply reflect the fact that SANE nurses have self-selected for additional training due to an interest in helping SA survivors, which may allow them to provide more sensitive care. Regardless of the reason, given mounting evidence to support the benefits of SANE care to patient and quality outcomes, SANE programs should be expanded and supported whenever possible.

## LIMITATIONS

The major limitation of this study was the reliance on retrospective, routine care data collected from the EHR. It is possible that some services or resources were offered and/or accepted by patients and simply not documented, and it is unknown whether one group was more likely to document than the other. Any service not documented was considered to not have been offered or received for the purposes of the analysis, which may have affected the outcomes if a large proportion of those services not documented were either not applicable or were actually provided. Additionally, there were five individuals who presented for SA twice during the study period. Each encounter was analyzed independently, but it is possible that the first ED experience impacted their choices during the second encounter. However, given that these individuals represented such a small proportion of the sample, it is unlikely that their inclusion significantly affected the results of the study. Due to the small sample size, the effects of survivor gender could not be fully explored, as gender differences between groups were not statistically significant. It is possible that the larger proportion of cisgender men in the non-SANE cohort affected outcomes, or that their gender affected either the likelihood of SANE care or their likelihood of accepting services, as men may be less trusting of their care team due to significant stigma.^24^^,^^25^

Furthermore, much of the study period included the COVID-19 pandemic, which may have impacted clinical documentation, availability of ED services, or willingness of patients to remain in the ED while waiting for results or referrals, although these should have impacted both SANE and non-SANE groups similarly. Lastly, this was a single-site study. While it is likely that the results are generalizable to other large, urban, adult EDs, further studies are needed to validate these results in other ED settings.

## CONCLUSION

This study revealed that sexual assault survivors in the ED who received care from a Sexual Assault Nurse Examiner were more likely to be offered and to accept standard-of-care SA services and resources. This may reflect the increased sensitivity and expanded skillset afforded by SANE training, or the ability of a dedicated SANE to work outside the time, space, and workflow constraints of a busy ED. While arranging for continuous SANE coverage in the ED can be logistically and financially challenging, it may not only benefit patient outcomes but allow hospitals to meet recommended quality metrics, which may be required by governing bodies or even tied to reimbursement in the value-based care model. Legislative support for SANE coverage should be expanded nationally, with parallel increases in funding to help hospitals implement continuous SANE coverage. This will positively impact the quality of care for survivors of SA, who may then be more likely to receive the services and treatment that they need after a traumatic event.
